# Traumatic Bladder Ruptures: A Ten-Year Review at a Level 1 Trauma Center

**DOI:** 10.1155/2019/2614586

**Published:** 2019-12-12

**Authors:** John Barnard, Tyler Overholt, Ali Hajiran, Chad Crigger, Morris Jessop, Jennifer Knight, Chad Morley

**Affiliations:** ^1^West Virginia University Hospitals, Department of Urology, Morgantown, USA; ^2^West Virginia University, School of Medicine, Morgantown, USA; ^3^West Virginia University Hospitals, Department of Surgery, Morgantown, USA

## Abstract

Bladder rupture occurs in only 1.6% of blunt abdominopelvic trauma cases. Although rare, bladder rupture can result in significant morbidity if undiagnosed or inappropriately managed. AUA Urotrauma Guidelines suggest that urethral catheter drainage is a standard of care for both extraperitoneal and intraperitoneal bladder rupture regardless of the need for surgical repair. However, no specific guidance is given regarding the length of catheterization. The present study seeks to summarize contemporary management of bladder trauma at our tertiary care center, assess the impact of length of catheterization on bladder injuries and complications, and develop a protocol for management of bladder injuries from time of injury to catheter removal. A retrospective review was performed on 34,413 blunt trauma cases to identify traumatic bladder ruptures over the past 10 years (January 2008–January 2018) at our tertiary care facility. Patient data were collected including age, gender, BMI, mechanism of injury, and type of injury. The primary treatment modality (surgical repair vs. catheter drainage only), length of catheterization, and post-injury complications were also assessed. Review of our institutional trauma database identified 44 patients with bladder trauma. Mean age was 41 years, mean BMI was 24.8 kg/m^2^, 95% were Caucasian, and 55% were female. Motor vehicle collision (MVC) was the most common mechanism, representing 45% of total injuries. Other mechanisms included falls (20%) and all-terrain vehicle (ATV) accidents (13.6%). 31 patients had extraperitoneal injury, and 13 were intraperitoneal. Pelvic fractures were present in 93%, and 39% had additional solid organ injuries. Formal cystogram was performed in 59% on presentation, and mean time to cystogram was 4 hours. Gross hematuria was noted in 95% of cases. Operative management was performed for all intraperitoneal injuries and 35.5% of extraperitoneal cases. Bladder closure in operative cases was typically performed in 2 layers with absorbable suture in a running fashion. The intraperitoneal and extraperitoneal injuries managed operatively were compared, and length of catheterization (28 d vs. 22 d, *p*=0.46), time from injury to normal fluorocystogram (19.8 d vs. 20.7 d, *p*=0.80), and time from injury to repair (4.3 vs. 60.5 h, *p*=0.23) were not statistically different between cohorts. Patients whose catheter remained in place for greater than 14 days had prolonged time to initial cystogram (26.6 d vs. 11.5 d) compared with those whose foley catheter was removed within 14 days. The complication rate was 21% for catheters left more than 14 days while patients whose catheter remained less than 14 days experienced no complications. The present study provides a 10-year retrospective review characterizing the presentation, management, and follow-up of bladder trauma patients at our level 1 trauma center. Based on our findings, we have developed an institutional protocol which now includes recommendations regarding length of catheterization after traumatic bladder rupture. By providing specific guidelines for initial follow-up cystogram and foley removal, we hope to decrease patient morbidity from prolonged catheterization. Further study will seek to allow multidisciplinary trauma teams to standardize management, streamline care, and minimize complications for patients presenting with traumatic bladder injuries.

## 1. Introduction

Urogenital tract injury occurs in approximately 10% of all abdominopelvic traumatic injuries with bladder rupture representing just 1.6% of these cases [1, 2]. Due to the structural protection from the bony pelvis, injury to the bladder is rare and usually associated with a high-impact injury [2, 3]. Bladder rupture can be classified as either extraperitoneal (EP) or intraperitoneal (IP). EP ruptures are more common and usually result from forceful impact to the anterior bladder [2, 3]. IP ruptures usually result from a rise in intravesicular pressure following an abdomino pelvic impact that causes rupture of one of the weaker points of the bladder such as the dome [2, 3].

The clinical presentation of bladder trauma patients may vary based on injury severity, but most patients have gross hematuria, difficulty with or painful voiding, and suprapubic pain [3]. Pelvic fractures are very common in patients with bladder injury. Pelvic fractures have been found to be associated with increased morbidity and mortality in bladder trauma patients, and identification of a pelvic fracture should raise clinical suspicion to assess for urogenital injury [4]. Although bladder rupture is rare, it is associated with significant patient morbidity and a mortality rate of approximately 22% [5]. In studies assessing mortality trends in patients with bladder rupture, it has been shown that there has been no improvement in the mortality rate in these patients over the last two decades [4].

The American Urological Association (AUA) has guidelines in place regarding the management of urogenital trauma [6]. The guidelines recommend formal retrograde cystography in stable patients with gross hematuria and/or a pelvic fracture or any other patient with signs and symptoms suspicious of bladder injury [6]. The guidelines state that urethral catheter drainage is the standard of care for both EP and IP bladder ruptures [6]. The AUA guidelines also recommend surgical repair for all IP bladder ruptures for prevention of peritonitis following intraperitoneal exposure to bladder contents [6].

Although guidelines recommend catheterization for all patients with bladder rupture, no specific recommendations regarding length of catheterization are in place. Additionally, there are no recommendations on ideal operative technique for management of intraperitoneal and/or complicated extraperitoneal bladder ruptures. The present study seeks to summarize the patients with bladder trauma at our center using 10 years of data. Herein, we assess the management of these patients including the impact of length of catheterization on bladder injuries and associated complications.

## 2. Methods

Following IRB approval, we performed a retrospective review of 34,413 blunt trauma cases using a database provided by the trauma registry at our institution during the last ten years (January 2008–January 2018). Patients included in the study all had an ICD-9 diagnosis of bladder injury. Patients were excluded if they were less than 18 years old.

Patient data collected included age, ethnicity, gender, BMI, mechanism of injury, type of injury, any associated additional injuries, and complications. We additionally assessed management of patients including imaging studies performed, catheterization length, and surgical modalities used. Two-tailed *T*-test was utilized via Microsoft Excel for comparison of means with *p* < 0.05 being considered significant.

## 3. Results

### 3.1. Demographic Data

Chart review identified 44 total patients with a bladder injury in the last ten years. Of these patients, the mean age was 41.8 years old, and the mean body mass index (BMI) was 24.8 kg/m^2^. Demographic data also showed that 55% identified as female, 45% identified as male, and 95% were of Caucasian ethnicity. Motor vehicle collisions (MVC) were the most common mechanism of injury representing 45% of total cases. Other common mechanisms of injury were falls (20%) and ATV accidents (14%) (Table 1).

### 3.2. Characterization of Injuries

Of the 44 identified injuries, 31 cases were classified as EP injuries (70.1%), and 13 were classified as IP injuries (29.5%). Pelvic fractures were present in 93% of cases, and gross hematuria was present in 95%. Additional solid organ injuries were present in 39% of cases. The most common additional solid organ injured was the lung, followed by the spleen and the brain. Interestingly, 18% of all bladder trauma cases had a concomitant second genitourinary organ injury with kidney being most commonly followed by the urethra.

### 3.3. Management

Formal retrograde cystography was performed in 59% of bladder trauma cases during the initial hospitalization. Of these patients, mean time from presentation at our facility to cystogram was 4 hours. All patients were catheterized following initial presentation and/or diagnosis of bladder injury. Operative management was performed for all intraperitoneal injuries as well as 35.5% of extraperitoneal injuries. Bladder closure in operative cases was typically performed in 2 layers with absorbable suture in a running fashion.

### 3.4. Outcomes

The intraperitoneal and extraperitoneal injuries managed operatively were compared, and length of catheterization (28 d vs. 22 d, *p*=0.46), time from injury to normal fluorocystogram (19.8 d vs. 20.7 d, *p*=0.80), and time from injury to repair (4.3 h vs. 60.5 h, *p*=0.23) were not statistically different between cohorts. Of the extraperitoneal injury patients who were managed operatively, the indications included planned orthopedic open fixation with permanent hardware to be exposed to the area of injury (*n* = 8), presence of a bony spicule within the bladder (*n* = 3), concomitant bladder neck or rectal injury (*n* = 1), and initial concern for intraperitoneal injury (*n* = 1).

In patients with catheter dwell time less than or equal to 14 days, the average time to fluorocystogram was 11.5 days. There were no complications in this cohort. In patients with catheter dwell time greater than 14 days, the average time to fluorocystogram was 26.6 days. Complications were present in 21% of these cases including urinary tract infection, DVT, and gross hematuria with clot retention (Table 2). Mortality during initial hospitalization was noted in 3 patients (6.8%).

## 4. Discussion

Over the past ten years, nearly all bladder injuries identified were associated with both gross hematuria and pelvic fractures. This finding falls in agreement with the AUA Urotrauma Guidelines that recommend formal bladder imaging for all patients with both findings [4]. However, only 59% of these patients received appropriate bladder imaging on initial presentation in the Emergency Department (ED) indicating an opportunity for improvement. Prior studies have also demonstrated weaker compliance with imaging recommendations of a CT cystogram or plain cystogram in patients with suspected bladder trauma. At a level 1 trauma center in Utah, only 24% of bladder injuries in a 15-year time span were diagnosed with cystogram or CT cystogram [7]. Additionally, they found that in bladder injury patients who only received standard CT imaging on presentation, 13% were missed or incorrectly diagnosed on initial presentation. and some cases were inappropriately operatively explored as a result [7]. Another study assessed patients with traumatic pelvic and acetabular fractures and found that only 47% of patients with pelvic or acetabular fractures that also had hematuria had a formal urologic evaluation on initial presentation [8]. These findings emphasize that although guidelines are in place for appropriate bladder imaging, they are not always being followed.

We additionally found that 39% of patients with bladder injuries had an additional solid organ injury. In another study of outcomes following genitourinary injury in United States (US) military members, it was found that genitourinary injury patients had a high incidence of concurrent traumatic brain injury as well as trends between GU injury and PTSD [9]. This emphasizes the significant morbidity of bladder trauma and the need for thorough evaluation for any additional injuries in all bladder injury patients.

Patients with catheterization longer than 14 days had a longer time to follow-up imaging as well as an increased complication rate. When assessing reasons for delayed catheter removal, it was often found that the delay was due to coordination of follow-up appointments rather than any medical indication for longer catheterization. The increased catheter dwell time and associated complications emphasize the need for sooner follow-up and imaging to allow catheter removal as soon as clinically indicated to minimize the risk of complications in these patients.

**Figure 1:**
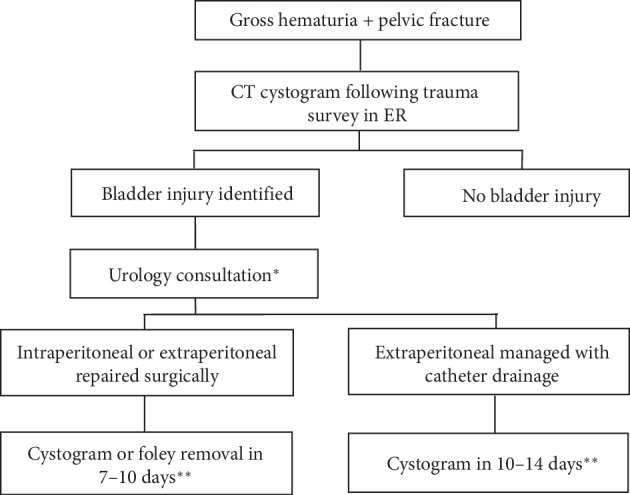
Revised institutional bladder trauma protocol. ^*∗*^Surgical repair is performed in all intraperitoneal injuries and extraperitoneal injuries involving the bladder neck, concomitant rectal or vaginal injury, exposure to orthopedic hardware (i.e., pelvic fixation), or nonhealing state after management with catheter drainage. ^*∗∗*^Revisions to our institutional protocol reflect standardization of length of catheterization based on the finding that catheterization less than 14 days resulted in decreased morbidity. Previously, there was no institutional consensus or protocol on length of catheterization, and it was highly variable.

Based on the findings of this study, our institution generated a standardized treatment algorithm which we plan to integrate into our institutional trauma protocols (Figure 1). House staff members covering the trauma service are provided with a written handbook containing all pertinent solid organ protocols to promote adherence to established protocols and homogeneity in treatment for all trauma patients in our high volume, level 1 trauma center. To date, length of catheterization after bladder injury was a metric that was not included or addressed at an institutional level or a national level as evidenced by no specific AUA Urotrauma recommendations. Given the results of our study, we aim to limit catheterization to less than 14 days for all bladder injuries given the higher complication rate observed for patients with catheter dwell times exceeding this metric.

## 5. Implications

The present study provides a 10-year retrospective review characterizing the presentation, management, and follow-up of bladder trauma patients at a tertiary care facility. Following this investigation, we introduced an institutional protocol for the management of bladder trauma patients hoping to increase adherence to Urotrauma Guidelines and to strictly define length of catheterization prior to initial follow-up cystogram. In patients with both gross hematuria and pelvic fracture, formal retrograde cystography should be performed in the emergency department following the primary trauma survey. If bladder injury is identified, urology consultation should be obtained promptly to guide management. Bladder catheterization should be performed, and if the injury is IP, operative management will follow. When possible, follow-up cystogram and urinary catheter removal should be performed within 10–14 days to limit the risk of complications in those patients who have a normal initial follow-up cystogram. Further study will seek to allow multidisciplinary trauma teams to standardize management, streamline care, and minimize complications for patients presenting with traumatic bladder injuries.

## Figures and Tables

**Table 1:** 10-year summary of contemporary bladder trauma.

Demographics	
Age	41.8 years (8–87)
Gender	55% female
Ethnicity	95% Caucasian/Nonhispanic
BMI	24.8 kg/m^2^ (16.6–35.3)
Mechanism	*N* = 44
MVC	45.5% (20)
Fall	20.5% (9)
ATV	13.6% (6)
Pedestrian vs automobile	9.1% (4)
Crush	6.8% (3)
GSW	2.3% (1)
Others	2.3% (1)
Injury type	
Intraperitoneal	29.5% (13)
Extraperitoneal	70.5% (31)
Pelvic fracture	93.1% (41)
Gross hematuria	95.5% (42)

**Table 2:** Summary of complications in patients with catheter dwell time >14 days.

Patient	Catheter dwell time (d)	Complication(s)	Treatment/outcome
1	38	Continuous urinary extravasation	Bilateral percutaneous nephrostomy placement
			Resolved after 11 weeks of maximal urinary diversion
2	43	Multiple UTIs/pyelonephritis	Neurogenic bladder requiring chronic intermittent catheterization
3	96	Urinary retention/UTIs	Neurogenic bladder requiring chronic intermittent catheterization
4	30	Lower extremity DVT	Resolved with anticoagulation therapy
5	34	Hematuria, calcification of catheter	Resolved with foley removal
6	27	Hematuria with clot retention	Resolved with bedside irrigation and foley removal
7	33	*E fecalis* UTI	Resolved with PO antibiotics

## Data Availability

The data used to support the findings of this study are available from the corresponding author upon request.

## Supplementary Materials

Supplementary Materials The supplementary data file is a table which has all of the demographic and clinical data for bladder trauma patients listed in a deidentified manner. Demographic data include age, gender, ethnicity, and BMI. The clinical variables include date and time of initial injury, mechanism of injury, concomitant injuries, and imaging studies. Additional data regarding length of catheterization, complications, and follow-up studies are also included.
